# Invasive Plants as Accumulators of Heavy Metals and Potentially Toxic Elements: A Review with Implications for Remediation

**DOI:** 10.3390/plants15071078

**Published:** 2026-04-01

**Authors:** Zorana Miletić, Miroslava Mitrović, Dimitrije Sekulić, Snežana Jarić, Natalija Radulović, Milica Jonjev, Pavle Pavlović

**Affiliations:** Department of Ecology, Institute for Biological Research Sinisa Stankovic, National Institute of the Republic of Serbia, University of Belgrade, Bulevar Despota Stefana 142, 11108 Belgrade, Serbia

**Keywords:** phytostabilization, phytoextraction, invasive plant species, trace elements, heavy metals

## Abstract

Invasive plant species frequently dominate contaminated ecosystems and are increasingly reported as accumulators of heavy metals and potentially toxic elements (PTEs). While this phenomenon is widely documented, its functional implications for contaminant dynamics and remediation-oriented management remain insufficiently synthesized. This review provides a comprehensive assessment of heavy metal and PTE accumulation in invasive plants across terrestrial and aquatic environments, with emphasis on tissue-specific partitioning, environmental context, and species-level variability. Based on field surveys, controlled experiments, and biomonitoring studies, we synthesize evidence for the accumulation of key elements (As, Cd, Cr, Cu, Ni, Pb, and Zn) in the roots and above-ground tissues of terrestrial and aquatic invasive plants. The available literature reveals consistent patterns of root-dominated sequestration in many terrestrial invaders, contrasted with enhanced shoot accumulation in fast-growing aquatic species. These patterns underpin divergent functional roles, ranging from contaminant stabilization in soils and sediments to conditional phytoextraction under managed harvesting. Rather than promoting invasive plants as remediation tools, this review frames them as unavoidable functional components of contaminated landscapes. We critically evaluate their advantages, limitations, and ecological risks, identify key research gaps, and propose a context-aware framework for interpreting invasive plant–PTE interactions in environmental management.

## 1. Introduction

Plant invasions and environmental contamination often intersect in the same disturbed landscapes. As a result, invasive plants established at degraded sites frequently become important components of contaminant retention, redistribution, and exposure pathways [1,2]. To clarify how these taxa function as accumulators of heavy metals and other potentially toxic elements (PTEs), this review compiles evidence on tissue concentrations across terrestrial and aquatic invasive plants and interprets root–shoot partitioning using the standardized uptake descriptors: bioconcentration factor (BCF) and translocation factor (TF) [2,3,4]. The synthesis highlights the core PTEs most consistently reported across contaminated soils, sediments, and aquatic systems (As, Cd, Cr, Cu, Ni, Pb, Zn) and evaluates how environmental conditions and plant traits influence accumulation patterns [5,6]. The resulting evidence is translated into a risk-aware management perspective by mapping empirical BCF/TF signatures onto the relevance of phytostabilization versus controlled phytoextraction, while accounting for feasibility constraints and secondary risks associated with contaminated biomass [1,2,4].

### 1.1. Heavy Metals and Potentially Toxic Elements as Persistent Remediation Targets

Contamination by heavy metals and potentially toxic elements (PTEs) remains a core challenge for environmental remediation, since these elements persist, accumulate, and can be remobilized when environmental conditions shift [3,7]. Across industrial, mining, urban, and agricultural landscapes, long-term anthropogenic contamination combines with ongoing emissions to maintain elevated contents in soils, sediments, and surface waters. These processes create heterogeneous contamination footprints that are difficult to treat at scale using engineering-only approaches [8,9]. In practice, contaminated sites are often managed under constraints of cost, land use, and ecological function, which has sustained interest in nature-based and plant-assisted remediation approaches [10,11,12].

In remediation contexts, risk and treatability are frequently governed less by total PTE contents than by speciation and bioavailability, because these determine mobility, plant uptake, leaching potential, and exposure pathways [13,14]. Site factors such as pH, redox dynamics, organic matter, ionic strength, and mineral assemblages can shift PTEs between more and less available pools. As a result, exposure levels may vary over time even where total PTE stocks remain stable [15,16,17]. In aquatic and riparian environments, sediments can function as both sinks and sources of PTEs, sustaining chronic exchange with vegetation and influencing spatial redistribution during hydrological disturbances [6].

Soil and sediment pollution with PTEs also behaves as a plant community-level selective force. Rather than acting as a uniform stressor, chronic contamination often functions as a selective ecological filter. This filter disproportionately constrains PTE-sensitive native species while enabling the persistence and dominance of stress-tolerant taxa, including many widespread invaders [6,18,19]. This framing is especially useful for remediation-oriented syntheses because it links long-term anthropogenic contamination to realistic vegetation outcomes at degraded sites—i.e., the plant community that establishes is often the plant community that mediates contaminant retention, mobilization, and surface stabilization [20,21]. From a remediation perspective, this means that contaminant management at degraded sites is inherently linked to the traits and accumulation behavior of the plant species that are able to persist under PTE stress.

Because conventional remediation (e.g., excavation, washing, intensive stabilization) can be costly, disruptive, and difficult to deploy across large, contaminated mosaics, plant-based strategies remain attractive as scalable complements, where rapid risk reduction, erosion control, and long-term stabilization are prioritized [22,23]. However, the feasibility of plant-assisted remediation depends on which species can establish and persist in contaminated substrates, which brings invasive plants to the foreground of many real-world contaminated landscapes [2,24].

### 1.2. Invasive Plants in Contaminated and Disturbed Environments

Invasive plants commonly dominate disturbed habitats that are also frequent remediation targets, including industrial wastelands, roadside and urban soils, riparian corridors, dredged sediments, and constructed or modified wetlands. In these environments, physical disturbance and resource pulses often intersect with chemical stress [2,24]. Establishment and growth studies indicate that early life stages can be strongly shaped by contamination and co-occurring resource conditions, influencing which taxa successfully recruit and persist under degraded site conditions [18,25]. These dynamics align with field observations that contaminated habitats often select for fast-growing, stress-tolerant species capable of maintaining biomass under multiple interacting stressors [26,27,28].

A consistent theme across case studies is that several invaders maintain growth, biomass production, or establishment under PTE exposure levels that reduce the performance of co-occurring natives [27,29,30]. This pattern supports the framing of contamination effects as an ecological filter acting on plant communities. In some systems, PTE exposure interacts with other biotic pressures, potentially altering defense allocation and biotic resistance in ways that may further shift competitive outcomes under contamination [31,32]. Such interactions matter for remediation, since they shape the stability of vegetation cover and the likelihood that planted or naturally recruiting communities remain functional over time [25,33]. In contaminated landscapes, where remediation success often depends on maintaining stable vegetation cover, these invasion-driven dynamics directly influence the feasibility and durability of plant-assisted remediation strategies.

Crucially, these plants can also modify contaminant behavior in ways that matter for management. Through rhizosphere processes, root exudation, litter inputs, and interactions with microbial communities, invaders may either enhance immobilization (supporting phytostabilization) or increase mobilization and near-field transport (raising exposure), depending on species traits and site chemistry [34,35]. In engineered or urban wetland settings, invasive macrophytes are increasingly discussed in relation to contaminant capture and removal strategies that combine vegetation performance with active biomass management [10,36].

A recurring synthesis challenge is whether invasive plants show consistent elemental signatures relative to natives under contaminated conditions. Evidence indicates that accumulation patterns are strongly species- and context-dependent, varying with contamination history, substrate type, life form, hydrological regime, and disturbance intensity [37,38]. This variability motivates species-resolved syntheses and standardized metrics that connect accumulation data to functional remediation outcomes rather than to invasion status alone [2,3].

In a controlled copper-gradient experiment, both the invasive *Reynoutria japonica* (syn. *Fallopia japonica*) and the native *Urtica dioica* increased tissue Cu with soil Cu, but *U. dioica* accumulated higher absolute Cu across most tissues, whereas *R. japonica* showed a stronger relative increase and higher tolerance thresholds, highlighting differences in detoxification and partitioning rather than consistently higher concentrations in the invader [39]. Congeneric clonal comparisons demonstrate that invaders can exceed natives for specific elements and organs under stress: *Sphagneticola trilobata* showed higher Pb concentrations than its native congener under Pb exposure and benefited more from clonal integration, indicating that invasion-related traits can enhance accumulation and redistribution under certain PTE regimes [40].

Field evidence from riparian habitats further shows that native species can equal or exceed invaders in accumulation-based indices: along the Sava River, native species displayed a significantly higher metal accumulation index for Li and Sr, while some invaders (e.g., *Amorpha fruticosa*) showed notable translocation capacity for Sr [41]. On ultramafic substrates, the invasive tree *Ailanthus altissima* exhibited lower Ni translocation and bioaccumulation factors than the native *Fraxinus ornus*, consistent with more efficient Ni exclusion rather than enhanced accumulation [42].

Collectively, these studies support interpreting the ecological significance of invasive plants in contaminated ecosystems as arising from a combination of dominance or biomass, tolerance, and tissue partitioning, rather than invasion status alone as a reliable predictor of higher PTE accumulation. This review does not promote the intentional deployment of invasive plants for remediation; rather, it examines how these species, which already dominate many contaminated sites, function as unavoidable components of contaminant dynamics and management decisions.

### 1.3. Accumulation of Heavy Metals by Invasive Plants: From Tolerance to Functional Outcomes

**Figure 1:**
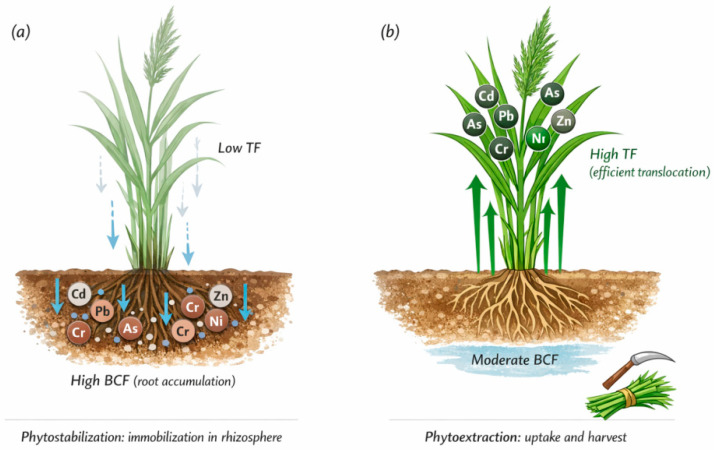
Conceptual illustration of phytoremediation strategies mediated by invasive plant species. (**a**) Phytostabilization, characterized by dominant accumulation of PTEs in the root zone and limited translocation to above-ground tissues (BCF > 1, TF < 1). (**b**) Phytoextraction, characterized by efficient translocation of elements to shoots and leaves (BCF > 1, TF > 1).

The remediation relevance of invasion dynamics becomes most apparent when attention shifts from species presence to patterns of PTE accumulation, tissue partitioning, and contaminant fate within plant biomass. For remediation, the central question is not only whether an invasive species tolerates PTEs, but also how it accumulates, partitions, and stores them across tissues (Figure 1). Species that retain PTEs predominantly in roots and rhizomes can reduce erosional losses and off-site transport, aligning with phytostabilization goals. In contrast, species that efficiently translocate PTEs to shoots may contribute to phytoextraction where harvesting and safe disposal are feasible [8,22,41]. In both cases, accumulation behavior is the key link between plant presence and remediation function, because it determines whether vegetation primarily stabilizes contaminant stocks in situ or supports controlled removal under management [3,9].

Evidence across aquatic and terrestrial systems shows that invasive macrophytes and riparian invaders can accumulate substantial PTE loads and, in some configurations, support contaminant removal through biomass harvest or management interventions [10,36,39]. At the same time, PTE accumulation can create secondary risks, including trophic transfer, litter-mediated redistribution, and practical challenges associated with handling contaminated biomass. Consequently, remediation relevance requires explicit risk-aware planning rather than reliance on accumulation metrics alone [23,43]. Management feasibility therefore depends on tissue distribution, biomass turnover, and the likelihood that accumulated PTEs remain immobilized versus re-entering mobile pools via decomposition or disturbance [44,45,46].

From an applied perspective, remediation planning benefits from distinguishing: (i) invaders that primarily stabilize contaminants in situ, (ii) invaders that mobilize contaminants and increase exposure risk, and (iii) invaders that accumulate and translocate PTEs in ways compatible with controlled removal—each class implying different management actions [22,23]. In some contexts, coupling invasive plant biomass management with complementary strategies (e.g., amendments, hydrological control, or engineered detention systems) may provide pragmatic risk reduction, but only when these species’ spread and downstream impacts are governed explicitly [47,48].

Despite a growing number of case studies, comparative syntheses linking invasion status to accumulation traits, root–shoot partitioning, and remediation feasibility remain fragmented across environments and taxa [33,49]. Generalizable insights therefore require integrating accumulation data with functional criteria—biomass production, tissue distribution, harvestability, and local risk context—to identify where invasive plants represent actionable remediation tools under controlled management versus where they primarily reshape PTE availability and complicate restoration trajectories [23,50,51].

### 1.4. Objectives and Scope of the Present Review

The present review provides a species-resolved, multi-regional synthesis of invasive plants as accumulators of heavy metals and PTEs, with explicit emphasis on accumulation patterns and functional outcomes relevant to remediation. The review centers on tissue-specific contents and root–shoot partitioning and interprets these patterns in relation to phytostabilization, phytoextraction, and risk-aware management of contaminated ecosystems. While the synthesis draws on studies from multiple continents, the underlying evidence base remains geographically uneven, as quantified in Section 9.3. Specifically, this review aims to achieve the following:

(1) Compile and evaluate evidence on heavy metal and PTE accumulation in invasive terrestrial and aquatic plant species;
(2) Identify general trends in root versus shoot metal partitioning across taxa and environments;
(3) Assess environmental and biological factors influencing accumulation capacity; and
(4) Discuss implications for phytostabilization and phytoextraction within a risk-aware management framework.

By centering on accumulation behavior and species-level evidence, this review complements mechanistic tolerance-focused literature and supports comparative analyses and evidence-based remediation planning in contaminated ecosystems.

## 2. Methodology of Literature Selection

This review is based on a comprehensive survey of peer-reviewed literature addressing heavy metal and PTE accumulation in invasive plant species. The literature search was designed to capture studies reporting empirical data on element contents in plant tissues, with particular emphasis on root and shoot or leaf compartments, across terrestrial and aquatic environments.

### 2.1. Literature Search Strategy

Relevant publications were identified through structured searches in major scientific databases, including Web of Science Core Collection, Scopus, and supplementary searches via Google Scholar. Searches were conducted using combinations of keywords related to biological invasions, metal contamination, and accumulation processes. Core search terms included the following:

invasive plant OR invasive alien species

AND

heavy metal OR trace element OR potentially toxic element

AND

accumulation OR uptake OR concentration OR content OR bioaccumulation

Additional searches were performed using species-specific queries based on the target invasive plants addressed in this review (e.g., *Solidago canadensis*, *A. altissima*, *R. japonica*, *Arundo donax*, *Myriophyllum spicatum*, *Tamarix* spp.). To ensure comprehensive coverage, synonymous taxonomic names and commonly used alternative genera (e.g., *Fallopia*/*Reynoutria*) were included where applicable.

The reference lists of key review papers and highly cited original studies were also screened to identify additional relevant publications not captured by database searches. The literature search covered publications from January 2000 to March 2026; the final database search update was performed on 7 March 2026 (records indexed in Web of Science/Scopus up to that date, including early-online/“online first” 2026 publications).

### 2.2. Inclusion and Exclusion Criteria

Studies were included in this review if they met the following criteria:

1. The plant species investigated was identified as invasive or alien in at least part of its reported range;
2. The study reported quantitative data on the contents of heavy metals or PTEs in plant tissues (roots, shoots, leaves, stems, or whole plants);
3. The study was conducted under field conditions, controlled pot experiments, mesocosms, or aquatic systems with documented element exposure;
4. The publication was peer-reviewed and available in English.

Since invasion status depends on the region and terminology varies across studies, we assigned “invasive/alien” status primarily based on the designation used by the authors of each included study for the study region (e.g., invasive alien species, non-native invasive, IAPS). To enhance transparency and consistency, we cross-checked species status against the Global Register of Introduced and Invasive Species (GRIIS) and/or the IUCN Global Invasive Species Database (GISD), and, where relevant for European contexts, the EASIN database.

Studies were excluded if they focused exclusively on the following:

1. Molecular, genetic, or transcriptomic responses to metal stress without reporting tissue-level accumulation data;
2. Physiological or biochemical markers (e.g., enzyme activity, oxidative stress indicators) in the absence of content measurements;
3. Phytoremediation trials using genetically modified organisms or engineered plant–microbe systems not representative of spontaneous invasive populations.

By applying these criteria, the review intentionally emphasizes functional accumulation patterns rather than mechanistic or molecular processes.

### 2.3. Study Selection and Screening Counts

A structured literature search was performed in Scopus (*n* = 768 records identified) and Web of Science Core Collection (*n* = 420 records identified). Google Scholar was used as a supplementary source for citation tracking and reference list screening (*n* = 16 new records identified). After merging and removing duplicates, *n* = 826 unique records remained and were screened by title and abstract. During this screening stage, records were excluded for the following primary reasons: non-plant taxa (*n* = 477), non-invasive plant focus (*n* = 72), non-PTE and/or contaminants outside the scope (*n* = 56), and review/methods-only records without primary tissue accumulation data (*n* = 18).

**Figure 2:**
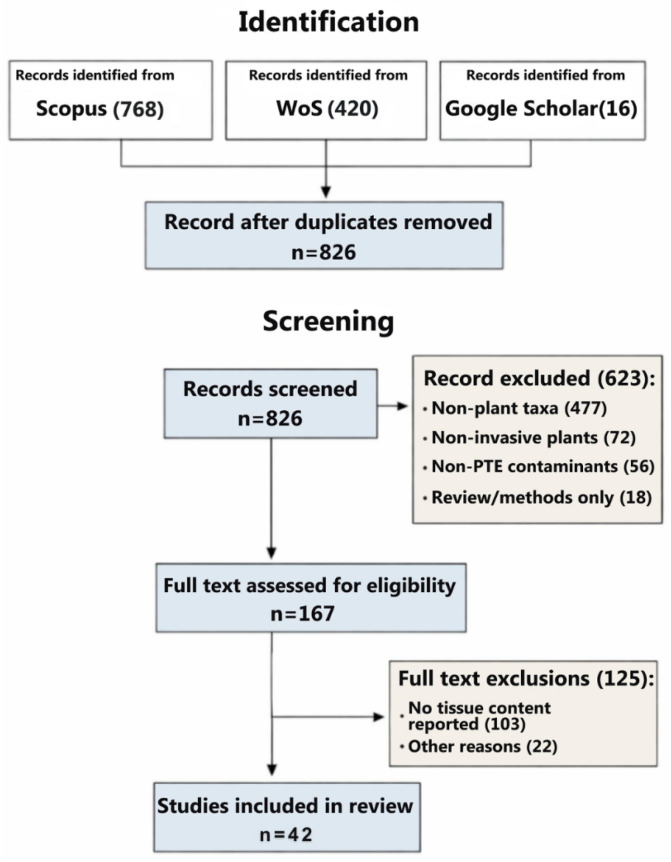
PRISMA-style flow diagram summarizing literature identification, screening, eligibility assessment, and inclusion for the qualitative synthesis.

Full texts were sought for the remaining records; full texts were assessed for eligibility (*n* = 167). At the full-text stage, articles were excluded primarily because tissue elemental concentrations (or extractable tissue content metrics) were not reported (*n* = 103) or because the available information was insufficiently reported for extraction or comparison (*n* = 22; e.g., tissue compartment not specified, values presented only qualitatively or in non-extractable figures, unclear units/basis, or overlapping datasets). The complete identification, screening, eligibility assessment, and inclusion process is summarized in a PRISMA-style flow diagram (Figure 2).

### 2.4. Elements and Environmental Contexts Considered

The review focuses on PTEs and metalloids most frequently reported in contaminated environments and invasive plant studies, including cadmium (Cd), lead (Pb), zinc (Zn), copper (Cu), nickel (Ni), chromium (Cr), and arsenic (As). Radionuclides and radioactive contaminants (e.g., uranium, thorium and isotope-specific radionuclides) were excluded a priori to maintain a consistent concentration-based synthesis across the PTEs most commonly reported in invasive plant tissue datasets used for BCF/TF interpretation (As, Cd, Cr, Cu, Ni, Pb, Zn). Inclusion of radionuclides would require a separate framework accounting for isotope/speciation reporting, radiological endpoints, and radioactive biomass handling/disposal considerations, which are not consistently documented across the invasive plant accumulation literature. Studies conducted in a wide range of environmental contexts were considered, including the following: contaminated agricultural soils, mine tailings and smelter-affected sites, industrial and urban brownfields, riverbanks and floodplains, and freshwater bodies and sediments colonized by invasive macrophytes. Both terrestrial and aquatic invasive species were included to enable cross-ecosystem comparisons of accumulation behavior.

### 2.5. Data Extraction and Synthesis Approach

For each selected study, information was extracted on plant species, geographic location, environmental setting, type and content of element assessed, and plant tissue compartments analyzed. Where available, data on soil or sediment PTE contents and relevant physicochemical parameters were also recorded to support contextual interpretation.

Given the heterogeneity of experimental designs, contamination levels, and analytical methods across studies, a qualitative and comparative synthesis approach was adopted rather than a quantitative meta-analysis, which would be inappropriate given the diversity of study designs and reporting formats. Emphasis was placed on identifying recurring patterns of PTE partitioning between roots and above-ground tissues, as well as consistent trends across species, environments, and contamination types.

The synthesis prioritizes species-level evidence and environmental relevance, with the aim of supporting interpretation in the context of remediation potential and ecosystem management.

## 3. Heavy Metals and Potentially Toxic Elements Considered

Heavy metals and PTEs are naturally occurring elements that become enriched in soils, sediments, and waters due to anthropogenic activities. Industrial processing, mining and smelting, urbanization, intensive agriculture, and waste disposal have contributed to sustained inputs of these elements into terrestrial and aquatic environments, resulting in persistent contamination across large spatial scales [3,52,53].

Among the elements most frequently reported in studies of contaminated ecosystems and invasive plant accumulation are Cd, Pb, Zn, Cu, Ni, Cr, and As. These elements recur across field surveys, greenhouse experiments, and remediation-oriented studies because they combine high environmental relevance with contrasting chemical behavior, mobility, and biological uptake pathways [28,38,54,55].

Unlike organic contaminants, heavy metals and metalloids are not subject to degradation and therefore persist in soils and sediments over extended timescales, where their bioavailability and ecological impact may fluctuate in response to changing physicochemical conditions. Consequently, contamination resulting from past anthropogenic inputs continues to influence present-day ecosystem functioning, vegetation composition, and contaminant exposure pathways, making these elements central targets for plant-assisted remediation and accumulation-focused research [2,36,52].

### 3.1. Environmental Relevance and Regulatory Concern

Cd and Pb are widely recognized as priority toxic elements in contaminated environments due to their persistence, toxicity at low contents, and limited biological function. Cd is relevant in remediation contexts because of its relatively high mobility in soils and its efficient uptake by plants, which facilitates transfer into food webs and increases ecological and human health risks [52,56,57]. Pb, while generally less mobile than Cd, accumulates strongly in surface soils and sediments near smelters, roads, industrial areas, and urban environments, where it can remain available to plants over long periods [53,58].

Zn, Cu, and Ni occupy an intermediate position between essential micronutrients and toxic contaminants. These elements play critical roles in plant metabolism at low contents but become phytotoxic when present at elevated levels in contaminated soils affected by mining, metallurgy, intensive agriculture, or waste disposal [53,59,60]. Their dual nutritional and toxic roles complicate interpretation of accumulation data, especially for invasive species that sustain rapid growth and high biomass production under moderate PTE exposure. In such cases, elevated tissue contents may reflect both enhanced uptake capacity and altered nutrient demand rather than toxicity alone [35,61].

Cr and As are of particular concern in industrial, mining, and sediment-associated contamination. Cr occurs primarily in trivalent and hexavalent forms, which differ markedly in mobility and toxicity, with hexavalent Cr posing substantial environmental and health risks due to its high solubility and bioavailability [52,62]. Arsenic contamination is frequently associated with mining residues, historical pesticide use, geothermal activity, and metallurgical processes, and its behavior in soils and sediments is strongly regulated by redox conditions, iron cycling, and microbial activity [53,63].

Because of their persistence, toxicity, and widespread occurrence, Cd, Pb, Zn, Cu, Ni, Cr, and As are subject to regulatory guideline values in soils, sediments, and waters across many national and international frameworks. Exceedance of these thresholds commonly triggers remediation requirements or land-use restrictions, making these elements central targets in studies evaluating plant-based remediation and accumulation potential, including those focused on invasive plant species [3,8,9,53].

### 3.2. Typical Behavior of Heavy Metals in Soils and Sediments

The behavior of heavy metals and PTEs in soils and sediments is governed primarily by physicochemical conditions that regulate speciation, mobility, and bioavailability, rather than by total contents alone. Parameters such as soil and sediment pH, redox potential, organic matter content, mineralogy, and cation exchange capacity exert strong control over PTE partitioning between dissolved, exchangeable, and solid-bound pools [53,64,65]. As a consequence, similar total PTE loads can result in markedly different exposure scenarios for plants depending on local environmental conditions.

In terrestrial soils, PTEs may occur as free ions in soil solution, be adsorbed to clay minerals and oxides, complexed with organic matter, or incorporated into secondary minerals. Cd and Zn generally display relatively high mobility under acidic conditions, whereas Pb is strongly associated with fine particles and organic matter, promoting surface accumulation and limited vertical migration [52,66,67]. Cu shows strong affinity for organic ligands, which can reduce its immediate bioavailability while promoting long-term retention in contaminated topsoils, whereas Ni mobility is often controlled by pH, clay content, and competitive interactions with other cations [64,68].

In aquatic systems, sediments act as dynamic reservoirs for heavy metals rather than inert sinks. Fluctuations in redox conditions associated with flooding, eutrophication, or plant root activity can induce PTE mobilization from sediment-bound fractions into pore water, facilitating uptake by aquatic and riparian vegetation [65,69]. This coupling between sediment chemistry and plant uptake is relevant in systems dominated by invasive macrophytes, where dense root and rhizome networks actively modify sediment structure and biogeochemistry.

Importantly, the presence of vegetation itself can alter element behavior in soils and sediments. Root exudation, rhizosphere acidification or alkalization, oxygen release, and interactions with microbial communities can shift PTE speciation and mobility at the soil–root interface [70,71,72,73]. In contaminated environments, these plant-mediated processes may either promote immobilization and stabilization or enhance mobilization and near-field transport, depending on species traits and environmental context. Such feedbacks underscore the need to interpret accumulation data in relation to both substrate chemistry and plant functional characteristics when assessing remediation potential.

### 3.3. Implications for Plant Uptake and Accumulation

Plant uptake of heavy metals and PTEs is governed primarily by the bioavailable fraction present in soils or sediments, rather than total element contents [74]. Bioavailability is shaped by PTE speciation, soil–sediment chemistry, and rhizosphere processes, as well as by plant functional traits that regulate root uptake, internal transport, and sequestration [59,75,76]. Consequently, plants growing in similarly contaminated environments may exhibit markedly different accumulation profiles depending on both environmental context and species identity.

In contaminated landscapes, invasive plants are typically exposed to complex mixtures of PTEs rather than to single-element stress. Interactions among co-occurring elements may influence uptake efficiency, translocation, and tissue-specific storage through synergistic or antagonistic effects at the root–soil interface and within plant tissues [77,78,79]. These interactions complicate direct comparisons across studies but also reflect realistic exposure scenarios at most contaminated sites targeted for remediation.

Accumulation patterns frequently differ between below- and above-ground tissues. Roots often act as primary sinks for elements such as Pb and Cr, where strong binding to cell walls and limited xylem transport constrain translocation and favor in situ immobilization. In contrast, elements such as Cd and Zn are more readily transported to shoots in many species, increasing their potential relevance for phytoextraction under controlled harvesting regimes [59,80,81,82]. These tissue-specific distribution patterns are central to evaluating whether invasive plants primarily stabilize contaminants within soils and sediments or contribute to their removal via biomass management.

From a remediation perspective, accumulation capacity alone is insufficient as a performance metric [83]. Effective evaluation requires consideration of biomass production, growth dynamics, tissue turnover, and the long-term fate of accumulated PTEs following senescence, litter deposition, or disturbance [22,35,59]. Invasive species that accumulate substantial PTE loads but rapidly recycle biomass may inadvertently promote local redistribution rather than net risk reduction if management is absent.

Accordingly, interpretation of accumulation data must integrate plant traits, environmental context, and management feasibility. This integrative view provides the foundation for distinguishing invasive species that function primarily as stabilizing agents, mobilization drivers, or candidates for controlled extraction, a distinction that is essential for remediation-oriented synthesis and decision-making.

#### Mechanistic Context Supporting Tissue Partitioning Patterns

Tissue concentrations and BCF/TF trends reflect the combined outcome of (i) rhizosphere bioavailability, (ii) membrane transport and xylem loading, and (iii) intracellular detoxification and compartmentalization. In many cases, uptake of non-essential metals (notably Cd) occurs via transport systems that primarily serve essential nutrients, including ZIP/ZRT-IRT-like transporters, NRAMP transporters, YSL transporters (metal–chelate transport), and HMA family ATPases, which contribute to long-distance transport and compartmental regulation [84,85,86]. Once inside tissues, continued accumulation under exposure is supported by detoxification processes such as chelation (e.g., phytochelatins/metallothioneins) and vacuolar sequestration, which reduce cytosolic toxicity while allowing retention in root tissues or storage in shoots, depending on species and element [49,84,85,86].

Rhizosphere processes can further modulate uptake by altering pH/redox conditions and complexation, including microbial transformations and root exudate-mediated mobilization or immobilization of metals [87,88]. In functional terms, these general processes help explain the recurring empirical signatures summarized in this review: strong root retention (high root contents, lower TF) is consistent with immobilization and binding/compartmentalization processes that constrain xylem transport (supporting phytostabilization interpretations), whereas elevated TF values imply more efficient xylem loading and shoot storage capacity, which is a prerequisite for managed phytoextraction when harvestable biomass is available [89].

## 4. Invasive Plant Species Reported as Metal Accumulators

A broad range of invasive plant species has been reported to accumulate heavy metals and PTEs across diverse environmental settings. Evidence from field surveys, controlled experiments, and biomonitoring studies demonstrates that PTE accumulation by invasive plants is not limited to specialized hyperaccumulator taxa but occurs across diverse lineages, growth forms, and ecological strategies [2,10,33]. Accumulation has been documented in terrestrial and aquatic invaders occupying agricultural soils, industrial and urban substrates, mining-affected landscapes, riverbanks, wetlands, and freshwater bodies, reflecting the broad environmental amplitude of many invasive species [8,49].

The diversity of invasive plants reported as PTE accumulators mirrors the heterogeneity of contaminated habitats they colonize. Invasive species differ widely in life history, growth rate, biomass production, and rooting strategy, resulting in substantial variability in tissue contents and element partitioning patterns reported across studies [82,90]. Consequently, accumulation behavior cannot be inferred solely from invasion status, but must be interpreted in relation to species traits and environmental context.

Importantly, many invasive plants reported as PTE accumulators are not introduced or managed intentionally for remediation purposes. Instead, they establish spontaneously at contaminated sites where their tolerance to disturbance and chemical stress enables persistence under conditions that limit native vegetation [22,33,91]. As such, these plants often represent the dominant or sole vegetation cover mediating PTE retention, mobilization, and surface stabilization at degraded sites, making their accumulation behavior directly relevant to remediation and risk management.

### 4.1. Major Taxonomic Groups and Life Forms

Studies examining PTE accumulation in invasive plants encompass species belonging to a wide range of taxonomic families and functional groups. Herbaceous species and grasses are well represented in the literature, largely due to their rapid growth, high reproductive output, and frequent dominance in disturbed and polluted environments [2,35]. Many of these species exhibit efficient uptake of PTEs from soils or sediments and may accumulate substantial contents in roots, shoots, or both, depending on species-specific traits and site conditions [75,92].

Woody invasive species, including shrubs and trees, have also been reported to accumulate heavy metals, although they remain comparatively less studied than herbaceous invaders. Their longer life spans and deeper or more extensive root systems allow prolonged interaction with contaminated substrates and integration of PTE uptake over time [22,93]. In many woody invaders, PTEs are retained predominantly in below-ground tissues or woody organs, a pattern that has implications for long-term stabilization rather than rapid contaminant removal.

From a life-history perspective, both annual and perennial invasive plants have been documented as PTE accumulators. Annual species may rapidly colonize contaminated sites and complete their life cycles under elevated PTE exposure, potentially contributing to short-term PTE uptake and redistribution through litter deposition and seasonal biomass turnover [18,20]. Perennial and clonal species, by contrast, may accumulate and retain PTEs across multiple growing seasons when extensive rhizome or stolon systems are present, thereby influencing long-term contaminant cycling within invaded ecosystems [94].

Aquatic invasive plants constitute a distinct and well-studied functional group within the accumulation literature. Submerged, floating, and emergent macrophytes have been widely reported to accumulate PTEs from both sediments and the water column, often displaying strong tissue-specific partitioning and close coupling with ambient contamination levels [46,95]. Their large surface area, direct sediment contact, and high biomass turnover make them relevant for studies of PTE bioavailability, redistribution, and potential removal in aquatic systems [20,96].

### 4.2. Frequently Studied Invasive Species and Geographic Patterns

Although element accumulation has been reported for a large number of invasive plant species, research effort is unevenly distributed across taxa and regions. A relatively small subset of these plants has been investigated repeatedly, often because of their broad geographic distribution, dominance in contaminated environments, or frequent occurrence at sites subject to monitoring or remediation [22,33,97].

Across terrestrial environments, frequently studied invasive species include herbaceous and woody taxa that commonly colonize contaminated soils, industrial land, and mining-affected areas. Species such as *S. canadensis*, *A. altissima*, *R. japonica*, *A. donax*, *Impatiens glandulifera* and *A. fruticosa* recur across field surveys and experimental studies assessing PTE accumulation in roots and shoots under contrasting soil conditions [98,99,100,101,102,103]. These species are often characterized by high biomass production, tolerance to disturbance, and the ability to persist under elevated PTE loads.

In aquatic and wetland systems, research attention has focused strongly on invasive macrophytes that dominate contaminated water bodies and sediments. Species such as *Eichhornia crassipes*, *M. spicatum*, and *Phragmites australis* have been extensively examined for their capacity to accumulate PTEs from both sediments and the water column, frequently displaying pronounced tissue-specific partitioning [24,104,105]. Because these species often form dense monospecific stands, their accumulation behavior has direct implications for contaminant retention, redistribution, and potential removal through biomass management.

Despite this growing body of species-level evidence, geographic coverage remains uneven. Most studies originate from Europe, East Asia, and parts of North America, regions with long histories of industrial activity and established environmental monitoring frameworks [2,8]. In contrast, invasive species occurring in Africa, South America, and Central Asia remain comparatively understudied, even where contamination pressure and invasion intensity are high.

## 5. Patterns of Metal Accumulation in Plant Tissues

**Figure 3:**
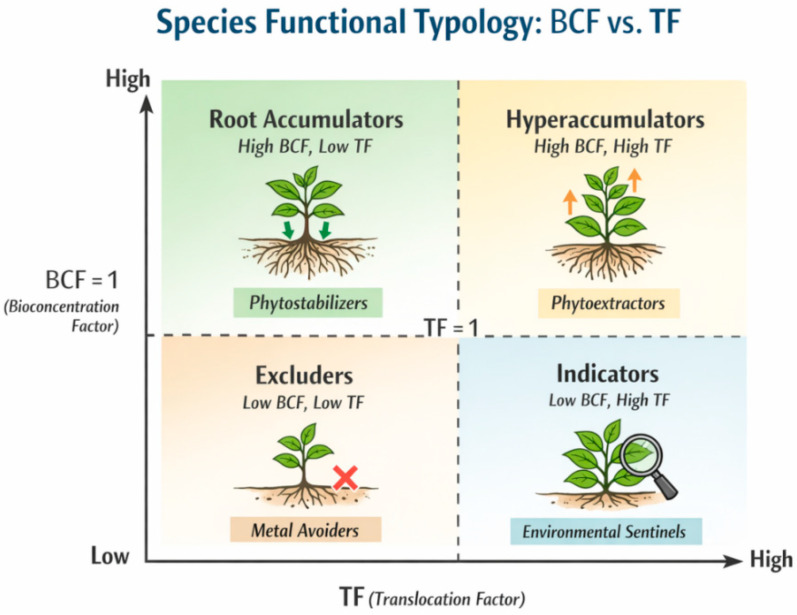
Conceptual classification of invasive plant species according to their functional behavior in PTE accumulation and translocation, based on the bioconcentration factor (BCF) and translocation factor (TF). Species are grouped into four functional categories: (i) phytostabilizers (high BCF, low TF), characterized by strong root retention and limited upward transfer; (ii) phytoextractors (high BCF, high TF), capable of both efficient uptake and shoot translocation; (iii) translocators (low-moderate BCF, high TF), exhibiting effective internal redistribution despite moderate root accumulation; and (iv) weak accumulators (low BCF, low TF), showing limited uptake and mobility. This typology integrates quantitative accumulation metrics to support the ecological interpretation of invasive plant roles in phytoremediation and element-specific management strategies.

Accumulation of heavy metals and PTEs in invasive plants is strongly tissue-specific, element-dependent, and influenced by plant functional traits. The synthesis presented in Table 1 and Table 2, together with the conceptual framework illustrated in Figure 3, demonstrates that these species cannot be treated as a homogeneous group of “PTE accumulators.” Instead, accumulation reflects distinct allocation strategies, with elements partitioned between roots, rhizomes, and above-ground biomass.

Figure 3 conceptualizes this species-functional typology by distinguishing stabilization-oriented species (high BCF, low TF), translocation-oriented species (TF ≥ 1 for mobile elements), and context-dependent accumulators whose behavior varies with environmental conditions. The quantitative data synthesized in this review confirm that these strategies are not random but follow recurring element-specific trends across terrestrial and aquatic taxa [10,41,42,106]. Understanding these tissue-level patterns is essential for interpreting whether invasive plants primarily contribute to contaminant stabilization, redistribution, or removal under remediation-relevant conditions.

### 5.1. Root and Below-Ground Accumulation

Root tissues represent the dominant compartment for PTE retention in most invasive taxa examined. This pattern is especially consistent for Pb and Cr, which exhibit limited mobility within plant vascular systems.

Extremely high root Pb contents were recorded in *Prosopis juliflora*, reaching 3366 mg kg^−1^ DW in contaminated soils [28], while *Pistia stratiotes* accumulated above 42,000 mg kg^−1^ DW in experimental conditions [107]. In woody invaders such as *A. altissima*, substantial Cr and Zn enrichment was documented in roots under experimental conditions [62,107], reinforcing a sequestration-dominated strategy.

Aquatic macrophytes demonstrated similar below-ground dominance. *Eichhornia crassipes* accumulated high contents of Pb, Cu, and Cr in root systems exposed to contaminated water columns (Table 1), while *M. aquaticum* reached Cr, Zn and Cd contents exceeding 2000 mg kg^−1^ DW in roots under heavy metal treatments [108,109]. In aquatic systems, direct contact with sediments and large absorptive surfaces amplify root-based accumulation.

Perennial herbs such as *S. canadensis* also displayed strong root retention for Pb and Zn [110]. Similar rhizome-centered immobilization was observed in *R. japonica*, where BCF values frequently approached or exceeded unity [111,112].

**Table 1:** Root accumulation of heavy metals and PTEs in invasive plant species.

Invasive Species	Root Content (mg kg^−1^ DW)	BCF	Other Elements Reported	Environmental Context	Study Type	Dominant Accumulation Pattern	Reference
*Acacia nilotica*	Cd 11.2	Cd > 1	/	Treatment with 10% NaCl	Experimental	Accumulation in roots	[113]
*Acacia saligna*	Pb 625 Cd 62	/	/	Pb, Cd treatment	Experimental	Root sequestration	[114]
*Ailanthus altissima*	Ni ~150 Cr 358 Zn 1348 Cu 75.7	Ni ˂ 1	Co	Ultramafic sites of central Italy; aqueous treatments	Field; Experimental	Root sequestration; Ni exclusion	[42,62,115,116]
*Alternanthera philoxeroides*	Pb 46–92 Cd 8–45 Cr 9–81	Cd, Pb > 1 Cr ˂ 1	/	Kolkata, India	Field	Pb and Cd phytostabilization	[117]
*Amorpha fruticosa*	Pb 80.9–954 Zn 335–4555 Cd 4.2–64.6 Cu 35.6–609	Pb ≈ 1 Zn, Cd and Cu ˂ 1	/	Organic amendments	Experimental	Pb phytostabilization; Zn, Cd, Cu excluder	[103]
*Arundo donax*	Cd 230 Cu 1630–1829	Cd <1 Cu > 1	/	Cd treatment, Cu treatment	Experimental	Root sequestration; Cu phytostabilization	[118,119]
*Eichhornia crassipes*	Cr 733–1258 Cu 23,387 Cd 1742 Hg 1762 Zn 2093 Pb 504 Ni 50.3	Cu, Cd, Ni > 1	/	Cr, Cu, Hg, Zn, Pb treatments; industrial effluents; Nile Delta (Egypt)	Field, experimental	Root sequestration	[120,121,122,123,124,125,126]
*Ludwigia peploides*	Zn 204–940 Pb > 500	Pb > 1000	/	Pb and Zn treatments	Experimental	Root sequestration	[127]
*Myriophyllum aquaticum*	Cd 367–2546 Cr 2291 Zn 8727 Ni 1742	Cd > 1	/	Heavy metal treatments	Experimental	Root sequestration	[108,109]
*Parthenium hysterophorus*	Zn 164	/	/	Zn treatments	Experimental	Root sequestration	[128]
*Phragmites australis*	Cr 1910	/	/	Cr treatments	Experimental	Root sequestration	[62]
*Pistia stratiotes*	Zn 434 Cu 93 Ni 90 Pb 42,862 Cd 3923	Zn > 1 Cu > 1000	/	Lake Mariut, Egypt; Al-Sero Drain—South Nile Delta, Egypt; Pb and Cd treatments	Field and experimental	Root sequestration	[107,129,130]
*Prosopis juliflora*	Pb 3366	Pb > 1	/	Pb treatments	Experimental	Phytostabilization	[28]
*Reynoutria japonica*	Pb 293 Cd 101 Zn 828 Cu 140	Cd ≈ 1 Zn > 1 Cu, Pb < 1	/	Brownfield, riparian ecosystem in Romania; Pb, Cd treatments	Field; Experimental	Pb phytostabilization	[111,112]
*Solidago canadensis*	Pb 38.5–789.5 Zn 221.1–2227	Pb > 1 Zn >1	/	Industrial and agricultural areas in Poland	Field	Pb and Zn phytostabilization	[110]
*Tamarix* spp.	Pb 73–316 As 661–1731	/	Cd, Al	Pb, As treatments	Experimental	Root sequestration	[131,132]

BCF—bioconcentration factor (root/soil or root/sediment); DW—dry weight.

### 5.2. Above-Ground Accumulation and Translocation to Shoots

In contrast to Pb and Cr, Cd consistently exhibited higher mobility across multiple taxa. This element-specific contrast is consistent with the broader tissue partitioning mechanisms summarized in Section 3.3, where Pb/Cr are frequently constrained by root cell-wall binding and limited xylem transport, whereas Cd (and often Zn) shows higher shoot mobility in many taxa [59,80,81,82]. Several invasive species demonstrated TF values ≥ 1 for Cd under field or experimental conditions (Table 2).

In *I. glandulifera*, Cd contents in shoots exceeded 1500 mg kg^−1^ DW, reflecting efficient translocation [102]. *Reynoutria japonica* similarly showed notable Cd transfer from roots to aerial tissues [111]. Experimental exposure of *P. hysterophorus* also revealed effective Cd and Zn redistribution to shoots under controlled contamination regimes [128,133].

Zn displayed intermediate behavior, often accumulating in both roots and shoots. In *A. fruticosa*, shoot Zn frequently exceeded 3000 mg kg^−1^ DW under experimental conditions [103], while *R. japonica* showed variable but substantial Zn contents depending on substrate conditions [112,134].

Aquatic species exhibited strong above-ground enrichment in some cases. *Eichhornia crassipes* accumulated Cu, Cr, and Cd in leaf tissues at levels relevant for biomass removal scenarios [120,121,122], whereas *M. aquaticum* demonstrated effective Cd, Ni and Zn redistribution within submerged tissues [108,109].

From a remediation standpoint, translocation to harvestable biomass is a prerequisite for phytoextraction. However, shoot accumulation alone does not ensure effective contaminant removal. Biomass productivity, harvesting feasibility, regrowth capacity, and safe disposal pathways all influence whether above-ground accumulation translates into effective risk reduction [22,82,83]. Moreover, in unmanaged systems, high PTE contents in leaves and stems may increase risks of trophic transfer or redistribution via litter decomposition.

**Table 2:** Shoot/leaves accumulation of heavy metals and PTEs in invasive plant species.

Invasive Species	Leaves/Shoot Content (mg kg^−1^ DW)	TF	Other Elements Reported	Environmental Context	Study Type	Dominant Accumulation Pattern	Reference
*Acacia saligna*	Pb 673–700 Cd 32	Pb, Cd ˂ 1	Cr, Ni	Pb treatments	Experimental	Accumulation in the seedling	[114,135]
*Acacia nilotica*	Cd 18.5	Cd ≈ 1	/	Treatment with 10% NaCl	Experimental	Phytoextraction	[113]
*Ailanthus altissima*	Zn 78.4–283	Zn ˂ 1	Cu, Mn	Coastal ridges in Romania	Field	Shoot sequestration	[115,136]
*Amorpha fruticosa*	Sr 55 Pb 6.7–110 Zn 58.5–3372 Cu 0.9–253	Sr > 1 Pb Zn Cu ˂ 1	Li, Cd	Sava River basin, Balkans; organic amendments	Field/Experimental	Sr phytoextraction Pb, Zn, Cu excluder	[37,103]
*Arundo donax*	Cd 191 Zn 59–170 Cu 130	Cd > 1 Zn > 1	As, Cr, Ni, Pb	Cd and Cu treatments; open-field experiment in Hungary	Experimental	Stem and leaf sequestration	[118,119,137]
*Eichhornia crassipes*	Cr 53–335 Cu 179 Cd 147	Cu ≈ 1 Cd < 1	/	Cr and Cu treatments; industrial effluents	Field; experimental	Leaf sequestration	[120,121,122]
*Elodea canadensis*	Zn 248	/	Cd, Cu, Cr, Ni, Pb, Mn, Fe	Streams and ponds in France	Field	Stem sequestration	[138]
*Elodea nuttallii*	Zn 442	/	Cd, Cu, Cr, Ni, Pb, Mn, Fe	Streams and ponds in France	Field	Stem sequestration	[138]
*Heracleum sosnowskyi*	Zn 380	/	Mn, As, Ni, Pb	Leningrad, Russia	Field	Leaf sequestration	[139]
*Hydrocotyle ranunculoides*	Ni 83.44 Zn 373 As 24.3 Cr 179 Cu 263	As ≈ 1	Cd, Co, Mn, V, Pb	Santa Bárbara stream, Brazil	Field	Stem sequestration	[140]
*Impatiens glandulifera*	Cd 276–1562	Cd ≈ 1	/	Cd treatments	Field; Experimental	Stem Cd hyperacumulator	[102]
*Ludwigia peploides*	Pb 264 Zn 739	Pb, Zn < 1	/	Pb and Zn treatments	Experiment	Leaves sequestration	[127]
*Myriophyllum aquaticum*	Cd 281–597 Cr 189 Ni 636 Zn 489	Cd > 1	/	Heavy metal treatments	Experimental	Leaf and stem sequestration	[108,109]
*Myriophyllum heterophyllum*	Cd 132	/	/	Cd treatments	Experimental	Leaf sequestration	[141]
*Parthenium hysterophorus*	Zn 252 Cd 854	Cd > 1	/	Zn and Cd treatments; EDTA additions	Experimental	Shoot sequestration	[128,133]
*Pennisetum setaceum*	Pb 157 Cu 392 Ni 321	Pb, Cu, Ni < 1	As, Mn, Zn	Cu–Ni mine in Selebi-Phikwe, Botswana	Field	Shoot sequestration	[142]
*Phragmites australis*	Cr 578	/	/	Cr treatments	Experimental	Stem sequestration	[62]
*Pistia stratiotes*	Zn 121 Cu 200 Ni 50 Pb 3867 Cd 624	Zn < 1 Cu > 1	/	Lake Mariut, Egypt; Al-Sero Drain—South Nile Delta, Egypt; Pb and Cd treatments	Field and experimental	Stem sequestration	[107,129,130]
*Prosopis juliflora*	Pb 1410	Pb < 1	/	Pb treatments	Experimental	Root sequestration	[28]
*Reynoutria japonica*	Zn 277–700 Cd 811 Pb 162	Zn > 1 Cd > 1	/	Urban and rural sites in Poland; brownfield, riparian ecosystem in Romania; Cd and Pb treatments	Field; Experimental	Cd and Zn phytoextraction	[111,112,134]
*Solidago canadensis*	Zn 48.2–969	Zn > 1	Pb	Industrial and agricultural areas in Poland	Field	Zn phytoextractor	[110]
*Tamarix* spp.	As 47–152	As < 1	Al	As treatment	Experimental	Leaves sequestration	[132]

TF—translocation factor (shoot–leaves/roots); DW—dry weight.

## 6. Factors Influencing PTE Accumulation in Invasive Plants

Accumulation of heavy metals and PTEs in invasive plants reflects interactions among element availability, plant traits controlling uptake and transport, and site-specific contamination characteristics. Accumulation capacity is not a fixed species trait; instead, it reflects interactions between substrate chemistry, rhizosphere processes, and plant growth strategies [18,75]. Understanding these interacting controls is essential for interpreting reported accumulation patterns and for evaluating the remediation relevance of these plants across contrasting ecosystems.

### 6.1. Soil and Environmental Conditions

Soil and sediment properties exert primary control on heavy metal bioavailability and subsequent plant uptake. Among these, pH is a dominant regulator of element solubility, with acidic conditions generally enhancing the mobility and plant availability of elements such as Cd, Zn, and Ni, while neutral to alkaline conditions promote sorption and precipitation reactions that reduce uptake potential [52,53,75]. Field studies conducted on contaminated floodplains, industrial soils, and mine-affected substrates consistently show that invasive plants growing under acidic or weakly buffered conditions exhibit higher root and shoot PTE contents than conspecifics colonizing neutral soils [98,143].

Organic matter content further modifies PTE behavior by providing sorption sites and complexing ligands. Depending on the PTE and environmental context, organic matter may either reduce bioavailability through strong complex formation or enhance mobility via dissolved organic compounds under fluctuating redox conditions [53,75]. Invasive plants capable of altering organic inputs through high litter production or root exudation can therefore indirectly reshape PTE speciation and availability within their rhizosphere, influencing both short-term uptake and long-term retention [20,76].

Soil texture and mineralogy also influence PTE partitioning. Fine-textured soils and sediments rich in clays and iron or manganese oxides typically exhibit greater PTE sorption capacity, constraining mobility but increasing near-surface accumulation [53]. In aquatic systems, sediments function as dynamic reservoirs rather than inert sinks. Redox fluctuations associated with flooding, eutrophication, or plant-mediated oxygen release can trigger PTE remobilization into pore water, facilitating uptake by invasive macrophytes rooted in contaminated sediments [144,145,146].

Importantly, vegetation itself modifies element behavior at the soil–root interface. Rhizosphere acidification or alkalization, oxygen release from roots, and interactions with microbial communities can shift PTE speciation and mobility locally, leading to either immobilization or enhanced mobilization depending on species traits and environmental context [59,147]. These plant-mediated feedbacks underscore the need to interpret accumulation data in relation to substrate chemistry rather than total element contents alone.

### 6.2. Plant-Related Factors

Plant functional traits strongly influence PTE accumulation and partitioning. Growth rate and biomass production are relevant in invasive species, where rapid establishment and high productivity may result in substantial total PTE uptake at the ecosystem scale even when tissue contents remain moderate [83,97,148]. This distinction between content-based and mass-based accumulation is critical for remediation assessments, especially in systems where erosion control and contaminant stabilization are prioritized.

Root system architecture plays a central role in determining both the magnitude and spatial distribution of PTE uptake. Invasive plants with extensive fine-root networks or clonal rhizome systems maintain prolonged contact with contaminated substrates, promoting interception and retention of PTEs in below-ground tissues [92,97,149]. In many woody and rhizomatous invaders, roots act as primary sinks for less mobile elements such as Pb and Cr, while more mobile elements such as Cd and Zn may be partially translocated to shoots.

Life-history traits further shape accumulation patterns. Annual invasive species may exploit contaminated niches rapidly and accumulate PTEs over short time frames, contributing to seasonal redistribution via litter inputs. Perennial and clonal species, in contrast, integrate PTE uptake over multiple growing seasons, supporting long-term retention or gradual cycling within invaded ecosystems [22,150]. Such differences have direct implications for remediation strategies based on stabilization versus removal.

### 6.3. Site-Specific and Regional Influences

Beyond soil and plant traits, site-specific conditions and land-use history exert strong controls on accumulation patterns. Urban soils are characterized by heterogeneous contamination associated with traffic emissions, construction activities, and diffuse industrial inputs, resulting in high spatial variability in plant PTE contents [151]. Industrial and smelter-affected sites typically exhibit elevated Pb, Zn, and Cu loads, while mining landscapes often combine extreme PTE contents with acidic conditions that enhance bioavailability [53,75].

Aquatic and riparian systems present additional complexity. Hydrological connectivity, sediment dynamics, and seasonal flooding strongly influence PTE availability and uptake by invasive macrophytes and riparian invaders [20,149,152,153]. Dense stands of these aquatic plants can both intercept PTEs from the water column and modify sediment chemistry, reinforcing feedbacks between vegetation structure and contaminant cycling.

Regional climatic conditions further modulate accumulation by influencing plant growth, soil processes, and hydrological regimes. Differences in precipitation patterns, temperature, and seasonality contribute to geographic variability in reported accumulation profiles, limiting the direct transferability of data across regions [35,149]. Consequently, accumulation patterns must be interpreted within their environmental and geographic context rather than extrapolated from isolated case studies.

Taken together, these interacting environmental, biological, and site-specific controls define the boundary conditions under which invasive plants accumulate heavy metals and PTEs. Integrating these factors provides the foundation for interpreting species-specific accumulation patterns and for distinguishing cases where invasive plants contribute to contaminant stabilization, mobilization, or controlled removal under remediation-oriented management.

## 7. Implications for Remediation

The accumulation patterns documented in Table 1 and Table 2 demonstrate that invasive plants occupying contaminated environments frequently exhibit species-specific and tissue-specific PTE partitioning profiles that directly influence their functional role in remediation contexts. Rather than behaving uniformly as either stabilizers or extractors, these species display a spectrum of strategies, ranging from strong below-ground retention with low translocation to efficient shoot accumulation of selected elements. While invasive plants may exhibit traits beneficial for contaminant stabilization or extraction, these functional attributes must be evaluated within broader ecological risk frameworks.

**Figure 4:**
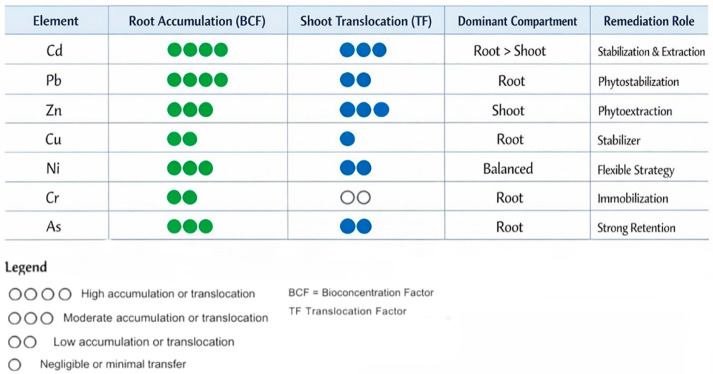
Conceptual matrix illustrating element-specific accumulation, translocation, and phytoremediation potential of invasive plant species. Root retention (BCF, colored in green) and shoot mobility (TF, colored in blue) are synthesized from published studies on PTE uptake in invasive species.

When accumulation data are synthesized across species and elements, a clear element-driven functional differentiation emerges, as illustrated conceptually in Figure 4.

The functional typology, synthesized in Figure 3 and mechanistically supported by the element–behavior matrix in Figure 4, provides a structured framework for interpreting invasive plant–PTE interactions beyond isolated content values. Importantly, accumulation patterns do not align strictly with invasion status or tolerance. Some invasive species tolerate high external PTE contents while limiting internal accumulation, whereas others accumulate substantial PTE loads without visible toxicity symptoms [49]. This decoupling underscores the need to interpret tissue-level data rather than relying on tolerance or growth performance as proxies for accumulation behavior.

Plant accumulation patterns provide a mechanistic basis for evaluating invasive plants within phytostabilization and phytoextraction frameworks. As summarized in Section Mechanistic Context Supporting Tissue Partitioning Patterns, element-specific partitioning reflects rhizosphere influences and intracellular detoxification/compartmentalization processes, and this mechanistic context is used here to interpret BCF/TF patterns within phytostabilization versus phytoextraction frameworks. Importantly, this review does not advocate the deliberate introduction of invasive species for remediation. Instead, it evaluates how these plants, already established in contaminated landscapes, influence contaminant retention, redistribution, and exposure pathways under controlled, risk-aware management. Recent synthesis work focused on mining-impacted environments has also discussed the potential role of invasive plants in phytoremediation, attributing interest to their high adaptive capacity and reported hyperaccumulation potential, while emphasizing the need for sustainable management during cultivation [154].

### 7.1. Potential Role in Phytostabilization

Phytostabilization aims to reduce contaminant mobility and exposure by retaining PTEs in soils and sediments and limiting their redistribution via erosion, flooding, or wind transport. Species that exhibit high root accumulation, low translocation factors (TF < 1), and extensive below-ground biomass are functionally aligned with stabilization processes.

Data synthesized in Table 1 and Table 2 indicate that several terrestrial and riparian invaders fall into this category. For example, *L. peploides* accumulates substantial Pb contents in roots while maintaining relatively limited shoot translocation [127]. Similarly, *P. juliflora* populations show strong root retention of Pb under experimental conditions [28]. In floodplain systems, *P. stratiotes* exhibits root sequestration of Zn under field conditions [129]. *Acacia saligna* demonstrates rapid root uptake but relatively constrained translocation for Cd under experimental conditions [114].

In waters with industrial effluents, *E. crassipes* shows elevated root contents of Cu, Ni and Cd with BCF ≥ 1, suggesting efficient accumulation [121,122]. Invaders such as *R. japonica* also exhibit substantial Cu and Zn root sequestration in riparian soils [112].

These traits enhance substrate stabilization through physical soil binding via dense root systems, reduced erosion and sediment transport, as well as through retention of less mobile elements (e.g., Pb, Cr) in below-ground compartments. Such stabilization is relevant in flood-prone riparian zones and mine tailings where mechanical redistribution poses major environmental risk.

However, phytostabilization does not eliminate contaminants. Elements retained in rhizosphere compartments remain sensitive to shifts in pH, redox status, hydrology, or land use in wetlands and floodplains [22,82,146,155]. Thus, invasive plant-driven stabilization should be interpreted as conditional containment requiring long-term monitoring rather than permanent remediation.

### 7.2. Potential Role in Phytoextraction

Phytoextraction depends on effective uptake and translocation of PTEs into harvestable above-ground biomass (both BCF and TF ≥ 1), combined with sufficient biomass yield to enable measurable PTE removal.

Among the species synthesized in Table 1 and Table 2, aquatic invaders demonstrate the clearest phytoextraction potential. *Eichhornia crassipes* and *P. stratiotes* frequently exhibit high shoot contents of Cu, with translocation and bioaccumulation factors > 1 reported in riverine systems [121,122,129,130]. *Myriophyllum aquaticum* similarly accumulates Cd in aerial tissues under heavy metal exposure [108,109]. In aquatic systems, removal efficiency is strongly linked to high biomass production and rapid growth rates.

Certain terrestrial species also show partial extraction potential. *Acacia nilotica* showed phytoextraction potential for Cd under experimental conditions [113], while *R. japonica* has phytoextraction potential for Cd and Zn under field and experimental conditions [111,112,134]. *Solidago canadensis* is shown to be a good phytoextractor of Zn in agricultural and industrial areas [110].

However, extraction efficiency is constrained by several factors: element-specific mobility (Pb and Cr generally exhibit limited shoot transfer), soil heterogeneity and mixed–PTE interactions, practical harvesting feasibility, as well as the risk of propagule dispersal during biomass removal. Even in aquatic systems, sustained phytoextraction requires repeated harvesting cycles and careful biomass disposal to prevent secondary contamination [156,157]. Therefore, phytoextraction is most relevant in moderately contaminated environments where invasive plants are already established, harvesting can be conducted safely, and invasive propagule dispersal can be effectively managed. Even under such conditions, phytoextraction should be considered a supplementary risk reduction strategy rather than a standalone remediation solution.

### 7.3. Ecological and Management Considerations

Any remediation-oriented interpretation of invasive plant accumulation must be embedded within a broader ecological risk framework. Although Table 1 and Table 2 demonstrate that several invasive species exhibit substantial PTE retention or translocation capacities, these functional traits cannot be evaluated independently of their ecological impacts and invasion dynamics.

Many of the species identified as effective accumulators—including *R. japonica*, *S. canadensis*, *I. glandulifera*, and *E. crassipes*—are widely recognized as aggressive invaders associated with biodiversity loss, competitive displacement of native vegetation, and structural alteration of riparian and wetland ecosystems [158,159]. Their dominance in contaminated landscapes often reflects disturbance-driven environmental filtering; yet enhancing their persistence or biomass production for remediation purposes may intensify invasion pressure beyond the contaminated footprint [160]. Consequently, any management action that indirectly promotes invasive growth must be critically evaluated against conservation objectives and existing invasive species regulations.

Field observations from floodplains and wetlands further illustrate that invasive species capable of strong root accumulation, such as *R. japonica*, *P. australis*, and *A. fruticosa*, often form dense clonal stands that stabilize substrates while simultaneously restructuring plant community composition [92,158,161]. In such cases, PTE retention and ecological dominance become tightly coupled processes. While substrate stabilization may reduce contaminant redistribution, it may also reinforce monoculture formation and suppress native succession. Thus, the functional benefit of contaminant immobilization must be weighed against long-term ecological simplification.

Management interventions themselves can introduce additional complexity. Harvesting operations intended to enhance phytoextraction, particularly for species such as *E. crassipes* or fast-growing riparian herbs, may inadvertently stimulate vegetative regeneration or propagule dispersal if not executed under strict containment protocols [156,159,160]. Clonal species with rhizomatous growth, including *Reynoutria* spp. and Phragmites australis, are especially sensitive to mechanical disturbance, which can fragment below-ground tissues and facilitate further spread. Therefore, remediation actions must be designed in a manner that does not amplify invasion intensity.

An additional management challenge concerns the fate of accumulated PTEs following biomass senescence or removal. In species that primarily stabilize PTEs below-ground, long-term persistence of contaminated root systems implies continued dependency on vegetation stability and site hydrology. In extraction-oriented scenarios, harvested biomass containing elevated contents of Pb, Cd, or Zn must be treated as contaminated material to prevent secondary pollution through decomposition, combustion residues, or leachate formation [20,82]. Without appropriate disposal pathways, PTE redistribution may offset initial gains achieved through accumulation.

Taken together, these considerations indicate that invasive plants should not be framed as remediation tools in isolation, but rather as context-dependent modifiers of contaminant dynamics within already invaded systems. Their ecological role is dual: they may enhance substrate stabilization and alter PTE partitioning, yet they simultaneously pose invasion risks that extend beyond contaminated sites. Effective integration of invasive plant accumulation into remediation frameworks therefore requires coordinated assessment of contaminant chemistry, invasion biology, regulatory constraints, and long-term land-use planning.

Practical remediation performance depends on harvestable biomass production, stand density, harvest frequency, and safe biomass handling. Across the studies included for extraction in Table 1 and Table 2, reporting is largely limited to tissue concentrations and BCF/TF metrics, whereas yield/biomass per unit area is inconsistently provided, limiting robust comparison of extraction rates (e.g., kg ha^−1^ per harvest) and time-to-target endpoints. In principle, removable mass per harvest follows a simple mass balance (removal = shoot concentration × harvestable biomass), but applying this consistently across studies would require assumptions not supported by the available literature. Therefore, the remediation implications discussed here are framed as risk-aware guidance: patterns consistent with root retention align more reliably with phytostabilization, whereas phytoextraction relevance requires, in addition to high translocation, demonstrable harvestable biomass and feasible biomass handling/disposal pathways.

## 8. Advantages and Limitations of Using Invasive Plants in Contaminated Environments

The frequent dominance of invasive plant species in contaminated ecosystems has generated increasing interest in their functional roles under conditions of chronic environmental stress. While invasive plants are primarily addressed within the context of biodiversity loss and ecosystem alteration, their repeated occurrence at polluted sites reflects a suite of traits that confer persistence, tolerance, and competitive advantage under elevated contents of heavy metals and PTEs. At the same time, any consideration of invasive species within remediation or management frameworks raises substantial ecological, regulatory, and ethical concerns. A balanced evaluation of both advantages and limitations is therefore essential for contextualizing accumulation data and avoiding misinterpretation of functional significance.

### 8.1. Advantages

A widely documented advantage of invasive plants in contaminated environments is their pronounced tolerance to abiotic stress, including elevated contents of PTEs such as Cd, Pb, Zn, Cu, and Cr. Numerous invasive species maintain physiological function, growth, and reproductive capacity at contamination levels that severely constrain or exclude many native taxa [18]. This pattern has been reported across terrestrial, riparian, and aquatic systems, including invasive herbs (*I. glandulifera*), clonal perennials (*R. japonica*), woody shrubs (*A. fruticosa*), trees (*A. altissima*), and aquatic macrophytes (*E. crassipes*, *M. spicatum*) [82,158,159].

Rapid establishment and high biomass production further enhance the functional relevance of invasive plants at polluted sites. Many invaders exhibit fast growth rates, efficient nutrient uptake, and extensive root or rhizome systems that facilitate early site dominance following disturbance. In contaminated soils and sediments, this rapid colonization can increase surface cover, reduce erosion, and limit the physical redistribution of PTE-bearing particles at unstable sites such as mine tailings, riverbanks, and industrial wastelands [21,162]. In this context, invasive plants may contribute indirectly to contaminant containment even when element uptake itself is moderate.

Another advantage is the ability of many invasive species to persist with minimal management input once established. Species already present at contaminated sites may provide continuous vegetation cover without deliberate planting, irrigation, or fertilization, which can be advantageous in large, remote, or economically constrained areas. For example, invasive riparian and wetland species frequently dominate PTE-contaminated sediments and maintain stable stands over multiple growing seasons, thereby contributing to site stabilization without active intervention [20,25].

The functional diversity of invasive plants also represents a potential advantage from an ecological perspective. Invasive taxa span a wide range of growth forms and habitats, including terrestrial herbs, grasses, shrubs, trees, and aquatic macrophytes. This diversity allows invasive plants to interact with contaminants across different compartments, including soils, sediments, pore waters, and water columns. Consequently, accumulation and stabilization processes associated with invasive plants operate across multiple ecosystem types rather than being confined to a narrow set of conditions.

Collectively, these traits help explain why invasive plants are consistently overrepresented at contaminated sites and why they feature prominently in studies of PTE accumulation and tolerance across diverse environmental contexts.

### 8.2. Limitations and Risks

Despite these apparent advantages, the use or functional interpretation of invasive plants in contaminated environments is subject to substantial limitations and risks. The most fundamental concern is ecological: invasive species are widely recognized as drivers of biodiversity loss, habitat homogenization, and altered ecosystem processes. Management actions that promote invasive plant persistence—whether intentionally or indirectly—may intensify invasion pressure and further suppress native vegetation in adjacent non-contaminated habitats [158,163].

Regulatory and ethical constraints further limit the applicability of invasive plants in remediation frameworks. In many regions, invasive species are subject to legal restrictions aimed at preventing their spread, and deliberate utilization may conflict with conservation policies or restoration objectives. Even when invasive species are already established, management interventions that enhance biomass production or longevity may be incompatible with broader biodiversity protection goals in protected or semi-natural landscapes.

Another critical limitation concerns the uncertainty of long-term outcomes. PTE accumulation by invasive plants does not equate to permanent risk reduction. PTEs retained in soils or root tissues remain within the system and may be remobilized following plant senescence, disturbance, hydrological change, or shifts in physicochemical conditions such as pH and redox potential. Several studies highlight that apparent short-term stabilization effects can be reversed under changing environmental conditions, challenging assumptions of sustained remediation benefit [22,67,164].

Biomass management poses additional challenges for species exhibiting above-ground accumulation or high tissue contents. Harvested invasive biomass containing elevated PTE loads must be treated as potentially contaminated material, requiring controlled handling and disposal. Inadequate management may result in secondary contamination through decomposition, inappropriate reuse, or dispersal of contaminated residues. This issue is especially acute for invasive plants capable of vegetative regeneration, where harvesting may inadvertently facilitate further spread if propagules are not effectively contained [20,165].

Finally, accumulation capacity alone is an insufficient indicator of remediation effectiveness. High tissue contents may coincide with low total PTE removal if biomass production is limited, while high biomass production may compensate for relatively low contents without achieving meaningful reductions in soil contamination. These trade-offs underscore the importance of evaluating invasive plants within an integrated ecological and management framework rather than interpreting accumulation metrics in isolation.

Taken together, the advantages and limitations associated with invasive plants emphasize the need for context-dependent evaluation. Invasive species may contribute to contaminant stabilization or redistribution in already invaded, highly disturbed environments, but their functional roles are conditional rather than universally beneficial. Framing these plants as opportunistic components of contaminated ecosystems—rather than as remediation tools per se—provides a more realistic and scientifically defensible basis for integrating accumulation data into environmental management discussions.

## 9. Research Gaps and Future Perspectives

Despite the expanding literature on heavy metal and PTE accumulation in invasive plants, substantial knowledge gaps remain that limit both synthesis and application. Existing studies provide valuable species- and site-specific insights, yet they frequently lack temporal depth, geographic breadth, and integration with ecological risk and management frameworks. Addressing these limitations will require a shift from descriptive accumulation reports toward coordinated, hypothesis-driven research that explicitly considers environmental context, plant functional traits, and long-term contaminant dynamics.

### 9.1. Understudied Species and Geographic Regions

Current understanding of PTE accumulation in invasive plants is strongly skewed toward a limited subset of taxa that are widespread, easily accessible, or historically associated with contaminated sites. Species such as *R. japonica*, *A. donax*, *E. crassipes*, and *P. australis* are repeatedly represented in the literature, whereas many regionally dominant invaders remain poorly studied despite their extensive distribution and ecological impact [97,106,166].

Geographic bias is equally pronounced. Most field-based accumulation studies originate from Europe, East Asia, and parts of North America, reflecting long-standing industrial activity, established monitoring networks, and research infrastructure. In contrast, large areas of Africa, South America, Central Asia, and the Middle East remain underrepresented, even though these regions host extensive mining, industrial contamination, and rapidly expanding invasive plant populations [97,166,167]. This imbalance restricts the ability to generalize accumulation patterns across climatic zones, soil types, and land-use histories. In the dataset extracted for Table 1 and Table 2 (*n* = 42), country-level locations were explicitly reported for *n* = 27 studies, with a strong clustering in Europe (*n* = 17) and Africa (*n* = 11) and minimal representation from other regions (Asia *n* = 1; South America *n* = 1; Oceania *n* = 0).

Future research should therefore prioritize invasive species that dominate contaminated landscapes outside traditionally studied regions and explicitly address regional drivers of PTE availability and plant uptake. Expanding geographic coverage will be essential for identifying whether observed accumulation patterns reflect universal functional traits or context-dependent responses.

### 9.2. Need for Long-Term and Field-Based Studies

A persistent limitation of the existing literature is the dominance of short-term experiments and controlled exposure studies. While pot trials and laboratory investigations provide mechanistic insight into uptake pathways and tolerance thresholds, they rarely capture the complexity of field conditions, where PTE availability, plant performance, and accumulation vary dynamically over time [22,59].

Long-term field studies remain scarce but are critical for understanding how invasive plants influence PTE cycling beyond initial establishment. Processes such as seasonal biomass turnover, litter decomposition, rhizome expansion, and root mortality can strongly affect the redistribution and stabilization of PTEs over multi-year timescales [149,168]. Without temporal replication, it remains unclear whether reported accumulation represents stable functional behavior or transient responses to local conditions.

Field-based studies are also essential for evaluating postaccumulation dynamics, including potential remobilization of PTEs under changing environmental conditions. Integrating long-term monitoring with detailed substrate characterization would substantially improve the ecological relevance and predictive value of accumulation data.

Extension of this synthesis to radionuclides (including uranium) also represents a relevant future direction but would require isotope-/speciation-aware reporting and performance metrics that differ from the concentration-based BCF/TF datasets synthesized here; related recent work often focuses on invasive plant biomass as engineered sorbents rather than tissue accumulation.

### 9.3. Strengths, Limitations, and Potential Sources of Bias

As with all narrative evidence syntheses, the assembled literature base is shaped by several sources of potential bias that influence interpretation.

First, database indexing and language patterns can favor studies published in widely indexed journals and English-language outlets, while access constraints can reduce the inclusion of otherwise relevant studies that are available only as abstracts or are not retrievable in full text (Figure 2).

Second, no formal study-quality scoring was applied because of substantial heterogeneity in experimental designs, contamination contexts, and reporting formats; consequently, reported accumulation values may reflect differences in replication, analytical protocols, and compartment definitions rather than biological contrasts alone.

Third, the evidence base shows clear geographic clustering. Within the final dataset extracted for Table 1 and Table 2 (*n* = 42), explicit country-level study locations were reported for *n* = 27 studies; these were concentrated in Europe (*n* = 17 studies with European sites) and Africa (*n* = 11; predominantly North Africa), whereas only single studies reported locations in Asia (*n* = 1) and South America (*n* = 1), and no included studies reported sites in Oceania. Several studies included multiple regions/sites, further emphasizing that geographic representation is uneven rather than globally uniform.

Fourth, taxonomic and element coverage is also uneven because research effort is concentrated on a subset of widely distributed invaders and the PTEs most frequently monitored across contaminated soils and waters.

Fifth, translation of concentration-based uptake metrics into operational remediation performance is constrained by inconsistent reporting of biomass and management variables (e.g., harvestable biomass per unit area, stand density/cover, harvest frequency, and post-harvest biomass handling) (see Section 7). As a result, comparable estimates of extraction rates per area (e.g., kg ha^−1^ per harvest) and time-to-target endpoints cannot be derived consistently across the included literature. Conceptually, the potentially removable contaminant mass per harvest follows a simple mass balance: Removal (g m^−2^) = C_shoot (mg kg^−1^ DW) × Biomass_shoot (kg DW m^−2^)/1000 (equivalent to kg ha^−1^ by multiplying g m^−2^ by 10), highlighting the key parameters that are often missing from primary studies. Accordingly, the remediation implications are framed primarily as risk-aware management guidance rather than time-to-cleanup predictions.

Collectively, these factors support interpreting BCF/TF trends as context-dependent signals and highlight the value of coordinated reporting (including biomass/yield and harvest regimes) and broader geographic coverage in future work.

### 9.4. Integration with Remediation Strategies and Management Frameworks

Although accumulation of PTEs by invasive plants is frequently reported, direct linkage between tissue contents and functional remediation outcomes remains limited. Many studies quantify PTE contents in plant tissues without assessing how these values translate into stabilization efficiency, extraction potential, or long-term risk reduction at the site level [22,50].

Future research should move beyond content-based metrics and explicitly integrate accumulation data with parameters such as biomass production, PTE mass balance, erosion control, and substrate stabilization. Distinguishing between plants that primarily immobilize contaminants in below-ground tissues and those that promote translocation to above-ground biomass is important for evaluating functional roles under phytostabilization versus phytoextraction scenarios.

Equally critical is the integration of accumulation research with invasion risk and regulatory frameworks. Invasive plants operate within legal and ethical constraints that differ markedly from those applied to native or deliberately introduced remediation species. Research efforts that ignore invasion dynamics, propagule pressure, and management feasibility risk producing results with limited practical applicability. Developing decision-support approaches that jointly evaluate accumulation capacity, ecological risk, and site-specific management objectives represents a key future challenge.

Overall, advancing the field will require a transition from isolated accumulation reports toward integrative, context-aware studies that explicitly address how invasive plants influence PTE dynamics over time and how this knowledge can be responsibly incorporated into contaminated land management.

## 10. Conclusions

Invasive plant species are increasingly recognized as frequent components of contaminated terrestrial and aquatic ecosystems, where they interact with heavy metals and PTEs in complex and context-dependent ways. This review synthesizes global evidence demonstrating that many invasive plants are capable of accumulating PTEs in their tissues, with substantial variability in accumulation magnitude, tissue partitioning, and environmental relevance across species and ecosystems. Invasive plants do not represent a uniform functional group but exhibit diverse accumulation strategies shaped by plant traits, environmental conditions, and contamination characteristics.

By focusing on tissue-level accumulation patterns, this review complements existing literature that emphasizes physiological or molecular tolerance mechanisms. The synthesis highlights that accumulation behavior—the distribution of PTEs between roots and above-ground biomass—is central to evaluating the functional implications of invasive plants in contaminated environments. Root-dominated accumulation may contribute to contaminant stabilization, whereas translocation to shoots may support controlled extraction under specific conditions. However, accumulation capacity alone does not determine remediation effectiveness and must be interpreted alongside biomass production, site stability, and ecological risk.

The findings further underscore that invasive plants should not be viewed as universal remediation tools. While they may contribute to stabilization or element redistribution in already invaded and highly disturbed environments, their use raises legitimate ecological, regulatory, and management concerns. Risks associated with further spread, impacts on native biodiversity, and handling of contaminated biomass necessitate cautious, site-specific evaluation. In most cases, invasive plants are best understood as opportunistic functional components of contaminated ecosystems rather than deliberate remediation solutions.

Finally, the review identifies key gaps in current knowledge, including limited geographic coverage, underrepresentation of many invasive taxa, and a scarcity of long-term field-based studies. Addressing these gaps will be essential for advancing understanding of invasive plant–PTE interactions and for developing integrative frameworks that link accumulation data with environmental management objectives. Future research that combines species-resolved accumulation data with ecological risk assessment and policy considerations will be critical for translating scientific insights into the responsible management of contaminated landscapes.

This review advances current understanding by synthesizing species-resolved evidence on tissue-specific accumulation of heavy metals and PTEs in invasive plants, with explicit consideration of how these patterns influence phytostabilization and phytoextraction potential. Rather than assessing accumulation capacity in isolation, we integrate environmental drivers, plant functional traits, and invasion risk to evaluate realistic management implications. By positioning invasive plants as conditional functional components of contaminated ecosystems rather than intentional remediation agents, this synthesis provides a risk-aware framework for interpreting accumulation data in applied and regulatory contexts.

## Data Availability

No new data were created or analyzed in this study.
